# The Degree of One Health Implementation in the West Nile Virus Integrated Surveillance in Northern Italy, 2016

**DOI:** 10.3389/fpubh.2017.00236

**Published:** 2017-09-05

**Authors:** Giulia Paternoster, Laura Tomassone, Marco Tamba, Mario Chiari, Antonio Lavazza, Mauro Piazzi, Anna R. Favretto, Giacomo Balduzzi, Alessandra Pautasso, Barbara R. Vogler

**Affiliations:** ^1^Istituto Zooprofilattico Sperimentale della Lombardia e dell’Emilia-Romagna (IZSLER), Brescia, Italy; ^2^Department of Diagnostic Medicine/Pathobiology, College of Veterinary Medicine, Kansas State University, Manhattan, KS, United States; ^3^Dipartimento di Scienze Veterinarie, Università degli Studi di Torino, Grugliasco, Italy; ^4^Servizio di Riferimento Regionale di Epidemiologia per la Sorveglianza la Prevenzione e il Controllo delle Malattie Infettive (SeREMI), Alessandria, Italy; ^5^Dipartimento di Giurisprudenza e Scienze Politiche, Economiche e Sociali, Università del Piemonte Orientale, Alessandria, Italy; ^6^Dipartimento di Scienze Politiche e Sociali, Università degli Studi di Pavia, Pavia, Italy; ^7^Istituto Zooprofilattico Sperimentale del Piemonte, Liguria e Valle d’Aosta (IZSTO), Turin, Italy; ^8^Department of Poultry Diseases, Institute of Veterinary Bacteriology, Vetsuisse Faculty, University of Zurich, Zurich, Switzerland

**Keywords:** One Health, evaluation, West Nile virus, integrated surveillance, zoonoses, Northern Italy

## Abstract

West Nile virus (WNV) is endemic in the Po valley area, Northern Italy, and within the legal framework of the national plan for the surveillance of human vector-borne diseases, WNV surveillance has over time been implemented. The surveillance plans are based on the transdisciplinary and trans-sectorial collaboration between regional institutions involved in public, animal, and environmental health. This integrated surveillance targets mosquitoes, wild birds, humans, and horses and aims at early detecting the viral circulation and reducing the risk of infection in the human populations. The objective of our study was to assess the degree of One Health (OH) implementation (OH-ness) of the WNV surveillance system in three North Italian regions (Emilia-Romagna, Lombardy, Piedmont) in 2016, following the evaluation protocol developed by the Network for Evaluation of One Health (NEOH). In detail, we (i) described the OH initiative (drivers, outcomes) and its system (boundaries, aim, dimensions, actors, stakeholders) and (ii) scored different aspects of this initiative (i.e., OH-thinking, -planning, -sharing, -learning, transdisciplinarity and leadership), with values from 0 (=no OH approach) to 1 (=perfect OH approach). We obtained a mean score for each aspect evaluated. We reached high scores for OH thinking (0.90) and OH planning (0.89). Lower scores were attributed to OH sharing (0.83), transdisciplinarity and leadership (0.77), and OH learning (0.67), highlighting some critical issues related to communication and learning gaps. The strengths and weaknesses detected by the described quantitative evaluation will be investigated in detail by a qualitative evaluation (process evaluation), aiming to provide a basis for the development of shared recommendations to refine the initiative and conduct it in a more OH-oriented perspective.

## Introduction

West Nile virus (WNV) is endemic in the Po valley area, Northern Italy. Within the legal framework of the national plan for the surveillance of human vector-borne diseases (WNV disease, chikungunya, dengue, Zika virus disease), WNV surveillance has over time been implemented in this area. Such surveillance is based on the transdisciplinary and trans-sectorial collaboration between regional institutions involved in public, animal, and environmental health. This integrated surveillance targets mosquitoes, wild birds, humans, and horses and aims at early detecting the viral circulation and reducing the risk of infection in the human population. Moreover, it is expected to enhance the surveillance efficiency and to save resources, by implementing targeted measures. To improve the surveillance sensitivity, data sharing mechanisms have been established among North Italian regions in 2016.

Considering the complex transmission cycle of WNV (see Identification of the System), a multi-disciplinary approach and a cross-sectoral collaboration between institutions involved in public, animal, and environmental health (i.e., a “One Health” approach) are better in obtaining knowledge on WNV circulation and subsequently prevent WNV transmission, as compared to a single-discipline and a uni-sectoral approach.

By following the evaluation protocol developed by the Network for Evaluation of One Health (NEOH), our evaluation quantitatively assessed how far the WNV surveillance system in 2016 is compliant with a One Health (OH) approach (“One Health-ness”), by considering three regions of Northern Italy (Emilia Romagna, Lombardy, and Piedmont). In detail, we quantified different aspects of the OH approach: the thinking and planning at the basis of the implementation of the surveillance system (“OH thinking” and “OH planning”); the commitment and involvement of actors and the infrastructure enabling a collaborative working and information sharing (“Transdisciplinarity and leadership” and “OH sharing”); and the individual and institutional gain in knowledge (“OH learning”) resulting from the initiative.

This quantitative evaluation of OH-ness, also in combination with other evaluation approaches (e.g., a process evaluation or a cost–benefit evaluation) enables the detection of strengths and weaknesses of the surveillance system and may thus be a basis for fine-tuning and implementing the initiative in a more OH-oriented perspective.

## Identification of the System

West Nile virus is a flavivirus maintained in a transmission cycle between wild birds and mosquitoes. While birds usually act as non-affected reservoirs, some mammalian species such as horses and humans may develop neurological disease (1). WNV was first described in 1937 after its isolation from a febrile woman in the West Nile region of Uganda but has been detected in Europe starting with an outbreak in horses and humans in the Camargue region, France, in 1962/63 (2). To date, WNV is endemic in several south European countries including Italy, where it first appeared in 1998 (3, 4) and then re-emerged in 2008 (5, 6). The transmission cycle of WNV is complex: it involves wild birds and mosquitoes, including their respective habitat, and humans and horses are at risk to develop (sometimes fatal) neurological disease. A holistic approach is thus needed to comprehend and influence the transmission system.

Over time, WNV-integrated surveillance was implemented by the regional health authorities in several regions of Northern Italy: Emilia-Romagna, Lombardy, Piedmont, Veneto, Friuli Venezia-Giulia. The rationale of the surveillance initiative is to use a multi-disciplinary approach to learn about all aspects of WNV circulation. The aim is the early detection of viral circulation in the species targeted by the surveillance activities, and subsequently reducing the infection risk in humans.

Infection in humans is mainly due to mosquito bites, but additional risks are related to infected blood transfusions and solid organ transplantations. Mitigating the risk of new WNV infections in the human population results in increased welfare (fewer individuals to suffer from West Nile disease) and consequently in reduced health care costs.

In our evaluation, we consider Emilia-Romagna, Lombardy, and Piedmont regions (study area), that cover the larger part of Italy’s Po Valley, a suitable breeding habitat for mosquitoes. The surveillance systems were implemented since 2009 in Emilia Romagna, and since 2014 in Lombardy and Piedmont. The evaluation will focus on 2016, when all three regions participated in the surveillance initiative with similar plans, and data sharing mechanisms among regions started (7–9).

All regions have the same sociocultural background and Italian is the main language. Additionally, the jurisdiction for all three regions is comparable for both animal health (veterinary national plan for arthropod-borne diseases in horses, national plans for wild and domestic birds, regional plans on wild animals) and public health (national plan to prevent transmission of WNV -and other arboviruses- by blood transfusion and organ transplantation).

Since WNV transmission is facilitated by mosquitoes as vectors, the transmission season for WNV overlaps with the activity of *Culex pipiens*, the main vector of WNV. Therefore, the European Centre for Disease Prevention and Control (1) assumes a theoretical transmission season for WNV from May to October. Accordingly, surveillance activities targeting wild birds, mosquitoes, humans, and horses are focused on that season (described in Section “Detailed Description of the Initiative and Scientific Background”).

## Description of the Initiative

### The Initiative within the System

#### Detailed Description of the Initiative and Scientific Background

To conform with the complex transmission cycle of WNV, involving birds, mosquitoes, and dead-end hosts, the initiative is comprised of four complementary parts: (i) active surveillance of avian target species, and passive surveillance of wild birds found dead (ii) active surveillance of mosquito target species, (iii) active and syndromic surveillance of horses with neurologic disease, and (iv) syndromic surveillance of human patients with neurologic disease.

All diagnostic tests are performed in public laboratories. Tests on avian species, horses, and mosquitoes are carried out by the official animal-health laboratories [Istituto Zooprofilattico Sperimentale (IZS)]. Tests on human samples (blood donations, organs, and samples collected from patients with neurological disease) are run in the reference laboratories of the Regional Health Services.

Avian target species (Eurasian magpies, *Pica pica*; carrion crows, *Corvus corone*; Eurasian jays, *Garrulus glandarius*) are shot within specific wildlife population control programs for agricultural pests, approved by the Italian Institute for Environmental Protection and Research. A fixed monthly number of these birds is collected, depending on their respective regional population size, density and distribution, and is submitted to the laboratory from each administrative province (from May to October in Emilia-Romagna, April to November in Lombardy, August to November in Piedmont). Brain, spleen, heart, and kidney samples are collected and analyzed using real-time RT-PCR, sequencing, and lineage determination. The sample is supplemented by passive surveillance of wild birds found dead.

Mosquito target species (*Culex pipiens, Cx. modestus*) are trapped from June to October. In Emilia-Romagna and Lombardy, the surveillance is carried out in the plain area, which is split into 11 and 20 km grid cells, respectively. In Piedmont, mosquitoes are trapped in plains and foothills, and surveillance areas (20 km grid cells) were selected by a risk-based approach. One entomological trap is placed in each cell or area under surveillance, for a total of 182 georeferenced stations. Collection is carried out fortnightly using CDC-CO2 dry ice-baited traps, BG-sentinel and gravid traps. Pools are analyzed using real-time RT-PCR, sequencing, and lineage determination.

Syndromic surveillance of neuroinvasive disease in dead-end hosts is carried out continuously throughout the year in all the three regions.

All horses with neurological signs are mandatorily notified to the veterinary authorities. From suspect cases, a blood sample is taken by the official veterinarian and tested for the presence of antibodies against WNV (IgM ELISA), and for the presence of virus specific RNA (real-time RT-PCR). In positive cases, this is followed by sequencing and lineage determination. In Lombardy, passive surveillance is supplemented by active surveillance on blood sera collected from horses in non-endemic provinces.

Syndromic surveillance of neuroinvasive disease in human patients is carried out continuously throughout the year in all three regions. All human patients with fever and one symptom of neuroinvasive disease [e.g., acute flaccid paralysis, acute polyradiculoneuritis (“Guillain–Barré syndrome”), aseptic meningitis, or encephalitis] are considered suspect cases of West Nile neuroinvasive disease (WNND). Plasma, serum, and cerebrospinal fluid are tested using real-time RT-PCR. In positive cases, sequencing and lineage determination are performed. This surveillance for suspect autochthonous cases of WNND is intensified in the period overlapping with mosquito activity in the regional territories, and particularly following the first viral detection at local level.

Adding to the complexity of the approach, not only the multi-species transmission cycle was taken into account when designing the surveillance system but also the different dimensions of life which should be targeted. The following dimensions were considered: populations (mosquito, bird, human, horse) to detect the infection rate; individuals (human, horse) to detect whether neurological signs may be due to a WNV infection; and tissues/organs to detect whether blood reserves or organs allocated for donation are infected with WNV.

Three trans-disciplinary working groups were created, in which experts of every health sector (animal, public, environmental) are represented:

(A) Animal health: IZSLER (IZS della Lombardia e dell’Emilia-Romagna, IZSLER, for Emilia-Romagna and Lombardy), IZSTO (IZS del Piemonte, Liguria e Valle d’Aosta, IZSTO, for Piedmont), Veterinary Services (Local Health Authorities—LHU); practitioners, horse owners, hunters, rangers.
(B) Public health: Public Health Services (Local Health Authority and Units), regional blood centers, reference laboratories for human diagnostics; physicians, hospitals.
(C) Environmental health: entomology centers (Centre for Agriculture and Environment, CAA, for Emilia-Romagna, Institute for Plants and Environment, IPLA, for Piedmont), IZSLER Virology and Epidemiology units collaborating with local Veterinary Officers for Lombardy.

The tasks to be carried out within this initiative were allocated to the named actors (defined as any individual, group, or organization who acts or takes part in the initiative and its context). The entomology centers (or local Veterinary Officers in Lombardy) are in charge of mosquito collection, while hunters with a specific permit and rangers collect the birds. The veterinary health institutions perform surveillance on animals and lab tests on animals and mosquitoes. The public health institutions are in charge of the surveillance in humans and testing of human samples. However, although each involved institution has specific tasks, they act for the common aim to early detect WNV circulation and reduce the risk of infection. The funding is provided by the respective Regional Health Services and, in Piedmont, also by research projects.

The initiative is guided by shared leadership between actors (veterinarians, medical doctors, biologists, and entomologists), who regularly meet in trans-disciplinary groups. Frequent regional (i.e., 3–4/year in Piedmont and Lombardy, 8–10/year in Emilia-Romagna) and plenary meetings are organized to allow actors to provide feedback. Joint activities among regions (i.e., sharing data on entomological traps at regional borders serving for surveillance for neighboring regions since 2016) are necessary for a complete coverage of the surveillance area, but also create team spirit and enhance communication.

Sharing and linking of information in inter-disciplinary groups within each region and communication between regions (meetings, periodic epidemiologic bulletin and updates on IZSLER website, email, phone calls) is well established. As described above, some information (i.e., lab test results on mosquitoes collected through entomological traps at regional borders) is shared among regions. In addition, in Lombardy, the results of the animal and mosquito surveillance are available online and updated daily.

Stakeholders of the initiative (defined as any individual, group, or organization who may affect, be affected by, or perceive themselves to be affected by the initiative) include human doctors, veterinary practitioners, hospital patients, and the general population. Seminars and educational activities are organized in order to increase the knowledge on the disease and involve stakeholders in surveillance activities, e.g., seminars and courses targeting official veterinarians and practitioners (especially horse veterinarians), medical doctors in hospitals and family doctors. The general population is involved through communication campaigns, websites, and informative leaflets and brochures. Reports of the activities are addressed to hunters and to individuals working with horses.

#### Drivers and Rationale

The initiative was started by the North Italian regions in response to recurring outbreaks of West Nile Disease and subsequent endemisation. Drivers for the initiative can therefore be assigned to four main categories: economical, emotional/psychological, environmental, and social:

(A) Economical: West Nile neurologic disease causes high healthcare costs for human and equine patients. Moreover, it determines financial damage in the form of DALYs (Disease Adjusted Life Years) for affected humans, in terms of loss of manpower for employers and of investments for owners of commercially used horses. Additionally, a continuous screening of blood donations from previously affected areas during the entire WNV circulation period is costly.
(B) Emotional/psychological: patients (human, horse) affected by neurologic disease are suffering. This suffering extends to the family and friends of the affected patients—especially in fatal cases.
(C) Environmental: possibly due to climate and environmental changes, mosquitoes—including the ones carrying WNV—have a higher chance of survival during winter (overwintering) leading to the establishment of WNV endemic areas in Northern Italy.
(D) Social: there is a lack of knowledge in the general population, regarding mosquito biology, their breeding habitats and their potential to carry disease.

The rationale of the initiative is to use a multi-disciplinary approach to early detect viral circulation in the species targeted by the surveillance activities, and the subsequent reduction of the infection risk in humans. Thanks to this early WNV detection, preventative measures can be applied in a more targeted way. The screening of blood donations from previously affected areas may be reduced to the active transmission season, starting with the first seasonal evidence of virus circulation. Additionally, public awareness campaigns may be intensified for populations at risk, once seasonal WNV transmission is detected.

### Theory of Change (TOC) of the Initiative

According to NEOH, the TOC is created for the actors to define the (long-term) goals of their initiative and the building blocks and resulting changes required to achieve these goals.

**Figure 1:**
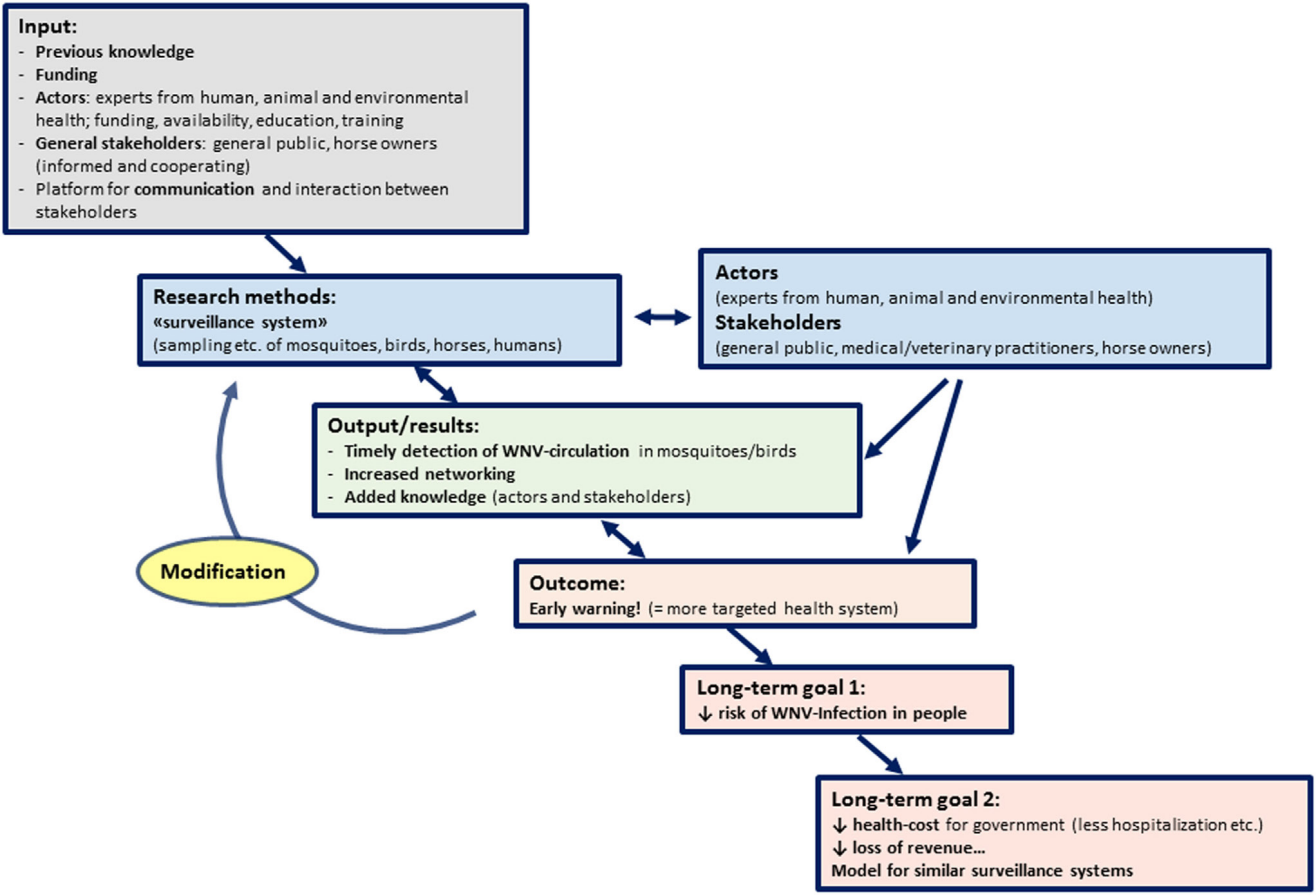
Pathway of Change representing the theory of change applied to the West Nile virus (WNV) surveillance in Northern Italy in 2016: building blocks, goals, and resulting changes required to achieve these goals.

For the presented initiative, these building blocks, necessary changes, and goals have been described in detail in the previous parts of this document. However, to align with the other manuscripts from this series, a summary, following the more schematic approach of a TOC, and the resulting graphical version of the TOC, the “pathway of change” (Figure 1), is provided.

West Nile virus is endemic to Northern Italy, being maintained in a transmission cycle between birds and mosquitoes. Humans and horses are accidental hosts and may suffer from a febrile illness to sometimes fatal neurologic disease. Transmission to humans may occur *via* bites of infected mosquitoes or the reception of infected blood or organ donations. The general population is insufficiently aware of the infection risks and thus poorly educated regarding strategies to prevent infection.

The basis for the initiative is the identification of appropriate actors with knowledge on WNV transmission, and of the stakeholders of the initiative. The funding of actors, including the support of networking infrastructure and other material and personnel, is essential. Additionally, stakeholders have to be compliant and respond to educational campaigns provided by the actors, by taking personal actions to prevent mosquito bites and maybe even by reducing breeding habitat.

The intermediate aim of the initiative is to prevent humans from being bitten by infected mosquitoes and from receiving infected blood transfusions or organ transplants. By preventing an infection, the risk of developing WNV disease will be reduced, and therefore the ultimate aim will be achieved: to decrease suffering and health care costs due to WNV disease.

To achieve these aims, experts from public, animal, and environmental health are working together to early detect seasonal WNV transmission in birds and mosquitoes, and to detect infections in horses and humans. After the first seasonal detection of WNV, blood and organ donations will be routinely screened, to decrease transmission risk. Additionally, educational campaigns for the general population may be carried out timely when seasonal infection risk increases resulting in a better-informed public that may act promptly and on their own initiative to prevent mosquito bites and to reduce breeding habitats.

As the initiative is ongoing, there will be a consolidation of networking among actors and an increase in expert knowledge on WNV transmission and disease, which will both feed back into modifying the approaches to achieve the intermediate and ultimate goals of the initiative. This results in a resilient system which can change over time to adapt to unexpected challenges, and according to the evolution of the epidemiological situation.

An indicator to measure the performance of the initiative may be a reduced annual incidence of human WNV cases. Additionally, an increased knowledge regarding WNV transmission and mosquito biology within the general population, maybe assessed by a questionnaire, could be a measure of successful performance.

### Assessment of the OH-Ness of the Initiative

In accordance with the evaluation protocol developed by NEOH, we first defined and later scored the five different aspects considered to be essential for a perfect OH approach: OH thinking, OH planning, Transdisciplinarity and leadership, OH sharing, and OH learning. Scores ranged from 0 (=no OH approach) to 1 (=perfect OH approach) and were allocated corresponding to the scoring key provided by the respective evaluation tool.

For the evaluation of OH thinking, different OH dimensions, i.e., geographical space and time frame of the initiative, the legislation it is based upon and which it may help to modify, the dimensions of life involved in the surveillance activity and the dimensions of life that the initiative may impact upon, were described. Also, we detailed the knowledge necessary for the creation and running of the initiative and knowledge resulting from it, the management of the initiative, the networking within and between groups and sectors, between actors and stakeholders as well as the influence of the economy upon the initiative (e.g., funding) and the impact of the initiative on the economy (e.g., reduction of health care costs). Scoring was based on different formulas considering the scales (e.g., local, national, global scale for the dimension “space”) we attributed to the different dimensions. Formulas for scoring included the relevance (i.e., the effect of the exclusion of the dimension on the initiative, and the effect of the initiative on the dimension itself), the balance among dimensions (i.e., equal weight), the highest scale and the number of scales.

When evaluating OH planning, we described all tasks to be carried out within the initiative and defined the stakeholders and necessary material, including additional personnel and funding. The tasks were further subdivided into responsibilities (e.g., the handling of an animal during blood collection) and matched with the professional skills of the involved personnel. A perfect match between responsibility and professional skill was scored as 1.0.

In the evaluation of Transdisciplinarity and leadership, scores were given according to the involvement of stakeholders, the effective involvement and integration of different disciplines, the collaboration among actors, the flexibility of the initiative, the degree of open-mindedness and presence of hierarchies.

To evaluate and clarify the processes of OH sharing, the different aspects of communication and sharing mechanisms were elucidated. Aspects included the basis on which potential actors were identified at the start of the initiative, how strongly they are involved in it, to what degree knowledge (data, methods, results) is shared between actors. Additionally, the resilience to change of sharing mechanisms was evaluated.

The evaluation of OH learning was based on multiple-choice questionnaires filled in by the actors of the initiative. Questionnaires were prepared in accordance to the ones provided by NEOH, translated into Italian and distributed to actors representing the different health sections (animal, public, environmental health) in each of the three regions. Scores were given as suggested by NEOH resulting in a mean score for all questionnaires.

## Results of the Evaluation

**Figure 2:**
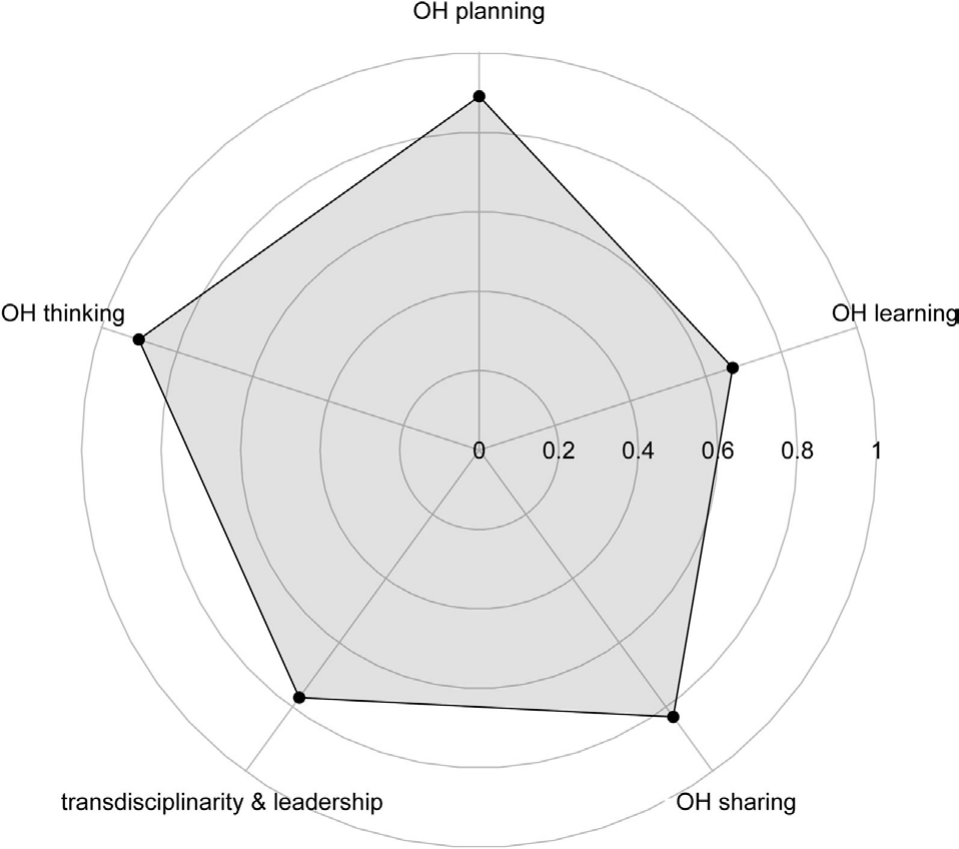
Spider diagram illustrating the degree of One Health (OH) implementation and the balance between the operational and the supporting means of West Nile virus surveillance in Northern Italy in 2016.

Five assessments were conducted, resulting in a mean score for each OH aspect considered. We reached high scores for OH thinking (0.90) and OH planning (0.89). Lower scores were attributed to OH sharing (0.83), Transdisciplinarity and leadership (0.77), and OH learning (0.67). The results are depicted in a spider diagram (Figure 2) with the surface area and shape illustrating the degree of OH implementation and the balance between the operational and the supporting means. We can observe a weakness in the infrastructure and supporting means for the initiative (OH learning and sharing infrastructure), namely critical issues related to communication, and learning gaps. In contrast, the operational aspects (OH thinking and OH planning) of the initiative are its main strengths, indicating a developed transdisciplinary team and comprehensive multi-dimensional approach. The results of the scoring of each OH aspect are detailed below.

The mean score for OH thinking was 0.90. Among the different dimensions assessed, space, knowledge, and networks obtained the highest mean score (=1.0), followed by dimensions of life (0.92), time (0.82), economy (0.80), management (0.77), and legislative dimension (0.72).

The initiative is implemented in a relatively small spatial (local, subnational, national) scale. However, as an operational aspect, the OH thinking of the system is influenced and has an impact both at a local level (e.g., provinces) and at a national level. Local, subnational, and state bounded knowledge is crucial for the creation and running of the initiative. Knowledge resulting from it is broad and can be categorized as local, subnational, and state bounded as well as universal, since it is added to the body of knowledge about WNV and disease surveillance and applies to countries with similar societal and epidemiological characteristics. Networking in the initiative is relatively complex, including interaction within and between work groups, within and between sectors (i.e., the animal and public health sector) and between stakeholders and the general population (trans societal). All scales were thus considered relevant.

Dimensions of life considered in the initiative include the gene, as well as the cell/organ, and population scale. In fact, humans, horses, wild birds, and mosquitoes are the target of the surveillance system (individuals, groups, populations). Samples of the above-named targeted species are tested for antibodies and/or gene sequences of WNV; after a first positive result in any species tested, human blood and organs are screened for WNV.

Time is of paramount importance in the planning of surveillance activities (e.g., organization, funding), therefore, surveillance plans are issued annually. However, the operational thinking required for their design (viral circulation in the geographical area, transmission season, vector and host distribution, etc.) can be independent from the time-frame of the plan implementation (e.g., 2, 5 years, etc.). Concerning the economic dimension, funding of the initiative is partly national (Ministry of Health) and partly regional. Both costs and benefits (e.g., reduction in welfare costs) of the initiative will impact at the regional and national level.

The management of the initiative is strategy-based and therefore rather broad and complex as compared to projects or work packages. Relevant legislation ranges from a rather low scale (operational rules, regional laws) to national and international regulations.

One Health planning was scored 0.89. A perfect match between responsibility and professional skill (score: 1.0) could be allocated to activities regarding the active surveillance on mosquitoes, surveillance of neuroinvasive disease in humans, laboratory tests on horses, wild birds, and mosquitoes, including species identification, laboratory tests on blood and organ donations, and on human suspects (WNND) samples, as well as activities regarding data sharing and communication. Surveillance on wild birds and passive surveillance on horses were considered as more critical and received a lower score, since such activities are partially carried out on a voluntary basis for lack of specific funding (Table 1).

**Table 1:** One Health (OH) planning.

Task	Match
Positioning of the entomological traps (active surveillance)	1.0
Collecting mosquito traps and transfer to laboratories (active surveillance)	1.0
Wild birds collection (trap/shoot) and transfer to the laboratories (active surveillance)	0.5
Passive surveillance on wild birds found dead	0.8
Passive surveillance in horses: reporting of suspect cases of WND (neurologic symptoms)	0.5
Passive surveillance in horses: sampling of suspect cases of WND (neurologic symptoms)	1.0
Active surveillance on horses	1.0
Laboratory tests on horses, wild birds, and mosquitoes incl. species-ID	1.0
Surveillance of neoroinvasive disease in humans	1.0
Laboratory tests on blood and organ donations, and on human suspects (West Nile neuroinvasive disease) samples	1.0
Data sharing and communication	1.0

**Overall score (mean) for OH planning**	**0.89**

*Description of the different tasks of the West Nile virus surveillance in Northern Italy in 2016. Scores were given for the match between necessary skills, possessed skills, and available personnel, material and/or infrastructure. A perfect match was scored as 1.0*.

Transdisciplinarity and leadership were given an overall score of 0.77. We considered the WNV transmission cycle, including the potential hazards for humans and horses (within this paragraph named the WNV “problem”) as well presented to the society (score: 1.0), with all actors and stakeholders involved in the initiative although not all efficiently engaged (0.8). Transdisciplinarity is necessary to solve the problem (1.0), being relevant to the health of people, animals, and environment. Disciplines, methods, and scales of analysis have an intermediate diversity (0.6). The initiative is broad and inter-sectoral (0.9), but there is a low involvement of the non-scientific community (0.1). There is a good balance across different disciplines and they work well together (0.9), however, gender is slightly biased toward male (0.7). Few cultural issues are considered to affect the problem (0.7). Regarding the integration, there is a good degree of interactions among actors of different disciplines (0.9), although this combination of disciplines cannot be considered as innovative (0.5). There is also a lack of formulation of a common OH objective covering all disciplines (0.2), with the initiatives aim being very “human health-oriented,” although such common objective could be a basis for knowledge integration. The project design has a very good flexibility at short, mid and long term (1.0). The initiative was considered as effective to contribute in detecting and solving the complex WNV problem (1.0). Although the management structure well supports the initiatives goal, with a good combination of disciplines/fields of expertise (0.9), leadership is task-oriented (0.1) with a limited open-mindedness (0.4) and rather static hierarchies. Different teams are working within the initiative and have a good level of cooperation (1.0) with fair inter-team relationships (0.8). Each team has clear objectives and their work is recognized at the organization level; however, they do not meet to discuss their effectiveness and how it could be improved. Competencies in the teams are appropriate to solve the problem (0.8). The initiative is very relevant to OH (1.0), and the problem is adequately translated to scientific questions (0.8) and has a solid scientific basis (1.0). Methods, collaboration, and integration fit the OH strategy (0.8) (Table 2).

**Table 2:** The Network for Evaluation of One Health questionnaire provided to assess transdisciplinarity and leadership was subdivided into different question complexes.

Question complex (no. of questions)	Score
Presentation of the societal problem within One Health (5)	0.94
Assessing broadness to further classify the initiative (3)	0.53
Assessing integration (10)	0.72
Assessing reflection, learning, and adaptation (3)	1.00
Assessing efficiency and effectiveness of the case study’s problem solving (2)	1.00
Assessing management, social and leadership skills (5)	0.35
Assessing team structure (well-structured vs. pseudo team) (8)	0.78
Actors and competencies (2)	0.80
Problem formulation, focus, goals, and criteria of success (6)	0.92

**Overall score (mean) for transdisciplinarity and leadership**	**0.77**

*For the West Nile virus surveillance in Northern Italy in 2016, the mean for each question complex was calculated*.

We attributed a score of 0.83 to OH sharing. In detail, stakeholders and actors are described in the regional laws, although a specific process of identification is not foreseen by the initiative (0.8). The overall involvement of stakeholders in the initiative, namely the personnel and hunters with a specific permit involved in the surveillance activities and the general population, was scored as good (0.7). Actors of the surveillance systems are highly involved in the initiative (0.9) through regular meetings, emails, reporting/writing of the epidemiological bulletins.

There is a high level of internal information sharing (0.9), with meetings, epidemiological bulletins, and reports available to all actors. Surveillance results are published and an online information sharing platform exists (10). External sharing mechanisms are good (0.7), with the online publication of activities, and surveillance results, and information campaigns during fairs and markets. Data and information sharing are funded within the mandatory activities of the institutions involved, and data sharing agreements are stated in the regional regulations.

Data quality is high (0.9), since data are compiled by dedicated staff/data analysts belonging to the different institutions. Bulletins are revised from all stakeholders. Appropriate (institutional) structures, databases, and backup systems ensure an appropriate data storage (0.9). Data accessibility is good (0.6), especially regarding the internal accessibility to maps and data. However, there is a limited accessibility to the general population, and the way it is made accessible (e.g., websites or brochures) varies by region. There is a good level of data sharing (0.7) and a high level of methods (0.9) and results (0.8) sharing among all actors. Methods, data, and results are continuously stored, resulting in an increased expertise over time (0.9); however, due to financial constraints, trained fixed-term personnel is “lost” in the process of time. Knowledge derived from the initiative is disseminated through congresses, seminars, meetings, and educational activities (0.9).

The level of resilience to change is high (0.9). Data are collected because of public (regional) funding: they are owned by the authority, always accessible and independent from changes to the system (e.g., change of laboratories performing the analysis).

**Figure 3:**
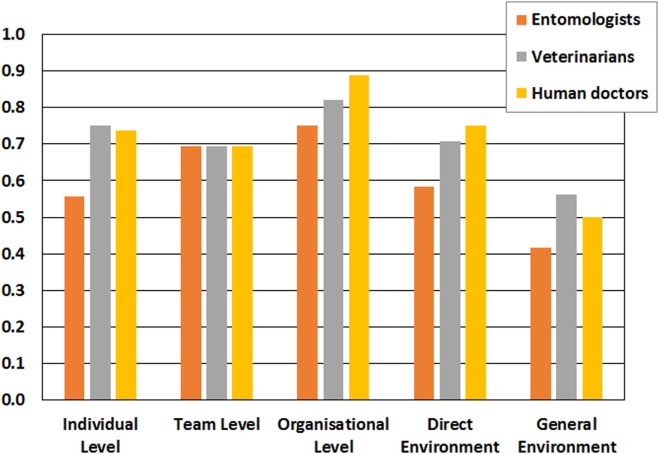
One Health learning of the West Nile virus surveillance in Northern Italy, 2016, considering different levels of learning and the supportiveness of the environment. The Network for Evaluation of One Health questionnaire was compiled by representative actors: an entomologist, a veterinarian, and a human doctor for each considered region. The bar plot states the mean score for each profession.

The multiple-choice questionnaires filled in by nine actors involved in the initiative (one entomologist, one veterinarian, and one medical doctor per region) gave a mean score of 0.67 for the OH learning. Entomologists gave a lower mean score (0.60), compared to veterinarians (0.70) and medical doctors (0.71) (Figure 3).

Although organizational, team, and individual learning levels are interconnected and influence each other, the highest mean score was obtained at the organizational level (0.81), followed by team (0.69) and individual (0.67) levels. Direct and general environments reached a mean score of 0.68 and 0.50, respectively.

These results show that organizations involved in the initiative (i.e., the different public institutions in the three regions) provide a high-level support for individual and team learning, and for their interplay within the context of the initiative. Specifically, this organizational support for learning reflects the existence of different resources to collect, store, and make available all existing information and knowledge to the individuals and teams involved. This enables possible modification of the organization’s underlying norms, policies, and objectives, based on the existing and acquired knowledge, providing resilience to the initiative. Differences in the mean scores assigned to organizational learning were observed among the three regions. Actors of Lombardy and Emilia-Romagna gave higher scores (0.73 and 0.72) compared to their colleagues in Piedmont (0.56), highlighting a possible weakness in the resources available for the OH learning in the latter region.

The team level learning was perceived as good (mean score 0.69). The teams participating in the initiative regularly meet for reporting results, and for sharing different perspectives and ideas aiming at obtaining the best view to inform decisions. Therefore, this result indicates a good interaction between the individual and the organizational learning of this initiative.

Individual learning was perceived as good by the actors, obtaining a mean score of 0.67. This score included both adaptive learning (i.e., how much the learning obtained was used to correct and improve existing procedures, competencies, technologies, and paradigms), and generative learning (how much the learning obtained was used to modify the organization’s underlying norms).

Finally, the context of the initiative (i.e., direct and general environment) was found to be scarcely supportive for learning. Specifically, the general environment (i.e., non-specific elements of the organization’s surroundings that might affect its learning like economic, technological, sociocultural, and others) obtained the lowest score (0.50). This could be due to the fact that society does not perceive governmental authorities as learning-oriented organizations.

## Further Developments: Process Evaluation

In addition to the quantitative OH-ness evaluation presented in this study, an ongoing qualitative evaluation (process evaluation) will provide data to confront to the OH-ness evaluation results.

The objective of the process evaluation is to detect strengths and weaknesses of how the initiative is planned and implemented using the opinion of “privileged observers” involved. In detail, the process evaluation is investigating (i) how the initiative is conducted, and the importance/sense given to it; (ii) the legal framework of the initiative as shared reference to fully adhere to.

Privileged observers’ opinions were collected in three focus groups, one for each region. Privileged observers are individuals having key roles in regional institutions involved in the WNV surveillance (regional Health Services; public health, animal health, and entomology centers). A maximum of eight participants attended each focus group, and they were selected among the actors involved in the initiative design and planning, and ideally represent the different disciplines involved (human and veterinary medicine, entomology).

Each focus group had a maximum duration of 90 min, was conducted by one moderator and one observer and recorded by an assistant. Specific questions on aspects considered important for the initiative were developed, as well as different documents to facilitate the discussion. These documents included a list of topics that should emerge with the questions, and prompt (solicitations) to make those topics emerge, if not spontaneously triggered by the questions. Fidelity questions were answered by participants using a flip board. Finally, general questions concerning the individual opinion on the initiative were submitted and answered in written form at the end of each focus group, in order not to influence the previous answers.

Preliminary results highlighted differences among Regions, mainly due to the different epidemiological situations (i.e., incidence of the disease in the human population). The critical points identified so far are related to communication and funding.

## Final Considerations

Considering the complexity of the transmission cycle of WNV (see Identification of the System), a OH approach seems necessary to guarantee the efficacy of any surveillance and control activity. In fact, knowledge on WNV circulation in all the species involved in the transmission cycle, and the subsequent informed decisions for its prevention, would unlikely be obtained through a single-discipline and uni-sectoral approach.

An integrated animal-human-vector approach to face WNV was adopted in several European countries (Austria, France, Greece, Italy, and UK), with activities varying according to the epidemiological scenario. Such approach may improve efficiency and save resources, thanks to the implementation of targeted control measures (11). For example, studies in Italy showed that the integrated surveillance had several advantages, both at national (12) and regional level, i.e., Emilia-Romagna region [(13); Paternoster et al., under review]. These included an increased efficiency in detecting infected blood units, the adoption of evidence-based preventative public health measures, and a reduction in health costs, thanks to a targeted strategy for testing blood units.

The evaluation protocol developed by NEOH and applied in the present study represents an innovative tool to assess the degree of OH implementation of a health-related initiative. To our knowledge, transdisciplinarity, collaboration, and communication aspects have not been specifically addressed in other studies. Researches so far mainly focused on performances or economic aspects of integrated WNV surveillance systems. In example, Kolmenakis et al. (14) performed an economic appraisal of public health management interventions adopted in Central Macedonia, Greece, to tackle the 2010 WNV outbreak. A study in the United States, assessed the cost-effectiveness of alternative WNV blood-screening strategies (15). In the French Mediterranean coast, Faverjon et al. (16) assessed that a multivariate surveillance system, which combines different data sources for WNV syndromic surveillance (e.g., reports of nervous symptoms in horses and wild bird mortality) had a major sensitivity and specificity in detecting outbreaks compared to approaches using data sources separately. Although this study underlines the importance of developing a more collaborative work between existing surveillance networks, no attempt has been made to evaluate the degree of inter-disciplinary collaboration. Similarly, Chaintoutis et al. (17) highlighted the usefulness of the surveillance on pigeons to determine WNV geographical spread and for early warning in Greece but made no specific assessment of the degree of implementation of a OH approach.

Our OH-ness evaluation confirmed the presence of an established multi-disciplinary approach and cross-sectoral collaboration (i.e., OH approach) in Northern Italy (Emilia-Romagna, Piedmont, and Lombardy regions). Surveillance is based on the collaboration between different regional institutions involved in public, animal, and environmental health, both at a regional and inter-regional level (see Results of the Evaluation). Several actors and stakeholders are involved and communicate within a complex operational and institutional network (see Detailed Description of the Initiative and Scientific Background).

The operational aspects (OH thinking and OH planning) are the main strengths of the initiative. Weaknesses were detected in the infrastructure and supporting means (OH learning and sharing infrastructure), namely critical issues related to communication, funding, and learning gaps. These results can be used to modify the system and improving its critical elements. Indeed, the OH-ness evaluation enables the detection of the strength of a multi-disciplinary approach and cross-sectoral collaboration of integrated zoonosis surveillance, providing insights that are useful for its improvement. Moreover, this standardized evaluation can be combined with other evaluations (e.g., process evaluation, cost–benefit evaluation) to measure the impact of the OH approach on the initiative. Further information can arise by comparing the OH-ness assessments of surveillance activities in different geographical areas, socio-economic contexts, or for different vector-borne diseases.

As regards our case-study, an ongoing process evaluation will provide a more detailed analysis of the surveillance planning and implementation. Evaluation results could (i) be the basis for developing shared recommendations, (ii) be used by Animal and Public Health decision makers at national or regional level, and (iii) provide insights on the efficacy of integrated health systems for zoonoses mitigation.

## Author Contributions

GP, LT, and BV conceived and designed the work and drafted the manuscript. MT, MC, AL, MP, and AP provided part of the data. AF, GB, GP, LT, and BV designed and carried out the process evaluation. All authors contributed to the analysis and interpretation of data, participated in the revision for important intellectual content, and approved the final version to be published. All authors agreed to be accountable for all aspects of the work in ensuring that questions related to the accuracy or integrity of any part of the work are appropriately investigated and resolved.

## Conflict of Interest Statement

The authors declare that the research was conducted in the absence of any commercial or financial relationships that could be construed as a potential conflict of interest.
